# Dentate Granule Cell Capacitance Is Stable across the Light/Dark Cycle

**DOI:** 10.1523/ENEURO.0213-25.2025

**Published:** 2025-09-18

**Authors:** Jose Carlos Gonzalez, Reagan L. Pennock, Asan F. Abdulkareem, Bryan W. Luikart, Jacques I. Wadiche, Linda Overstreet-Wadiche

**Affiliations:** ^1^Department of Neurobiology and McKnight Brain Institute, University of Alabama at Birmingham, Birmingham, Alabama 35294; ^2^Department of Molecular and Systems Biology, Geisel School of Medicine at Dartmouth College, Hanover, New Hampshire 03755

**Keywords:** circadian, dentate gyrus, excitability, integration, intrinsic

## Abstract

The plasma membrane acts as a capacitor that plays a critical role in neuronal excitability and signal propagation. Neuronal capacitance is proportional to the area of the cell membrane; thus it is often used as a measure of the cell size that is assumed to be relatively stable. Recent work proposes that the capacitance of dentate granule cells (dGCs) and cortical pyramidal cells changes across the light/dark (LD) cycle in a manner that alters synaptic integration. We addressed this potential change in capacitance using a large dataset of dGC recordings from adult male and female mice across the light cycle. Our data show that daily changes in the membrane time constant result from fluctuation in membrane resistance rather than capacitance. We also confirm the ability to resolve changes in neuronal capacitance induced by altering dGC membrane area via acute axotomy or genetically induced overgrowth using either voltage-clamp or current-clamp approaches. Our results demonstrate that the capacitance of dGCs remains stable over the LD cycle and that daily changes in the membrane time constant and excitability are mediated by fluctuations in membrane resistance.

## Significance Statement

There is increasing evidence that neuronal excitability fluctuates across the light cycle in many brain regions, including the dentate gyrus. Here we demonstrate that the capacitance of dentate gyrus excitatory neurons remains stable across the light cycle and that daily changes in granule cell-intrinsic excitability are associated with changes in membrane resistance rather than capacitance. These results support the conventional view that capacitance is a largely stable property reflecting the static nature of neuronal macroanatomical structure.

## Introduction

The time constant of neuronal membranes contributes to the speed of membrane potential changes that govern the efficiency of synaptic integration. It is a product of the resistance and the membrane capacitance. In turn, membrane capacitance depends on the dielectric constant of the phospholipidic bilayer, the membrane area, and the distance between conductive milieus. Traditionally, the plasma membrane thickness and composition has been viewed as a biological constant with an estimated value of ∼1 μF/cm^2^ (Gentet et al., 2000). Hence experimental measures of whole-cell capacitance are commonly used as an estimation of the neuronal size, and there are many technical discussions about optimal approaches for measuring capacitance in voltage-clamp and current-clamp recordings (Golowasch et al., 2009; Taylor, 2012). In voltage clamp, capacitance is typically estimated by measuring charge accumulation from current transients in response to small voltage steps (the test pulse). However, the accuracy of this approach for neurons with complex morphology is limited since the current transients provide a measure of capacitance from the “well clamped” portion of the cell. In current-clamp recordings, exponential fits of voltage responses to small current injections provides more accurate measurements across diverse cell sizes and morphologies. Yet, regardless of the approach used to measure capacitance, it is generally assumed to be a stationary parameter that reflects the relatively stable size of a given neuron.

Recent work reports that the membrane capacitance in two types of excitatory neurons exhibits a large oscillation across the time of day with a robust effect on synaptic integration (Severin et al., 2024). The degree of change, on the order of 60–100%, is associated with changes in time constant but no change in membrane resistance. Despite the growing literature on circadian regulation in membrane excitability, most variation is associated with changes in membrane resistance rather than capacitance (Paul et al., 2020). For example, we reported variation in dentate granule cell (dGC) synaptic integration across the light cycle associated with changes in the membrane time constant measured by either EPSPs or current injections (Gonzalez et al., 2023). The mechanism involves a 24 h cycle of G-protein–coupled inwardly rectifying K^+^ (GIRK) and Na^+^ (NaLCN) currents that contribute to basal membrane resistance. Here we use our large dataset of dGC recordings to evaluate the potential role of membrane capacitance in the daily cycle of membrane time constant. To that end, we used current-clamp recordings similar to Severin et al. (2024) to estimate capacitance in neurons with complex architecture (Golowasch et al., 2009; Severin et al., 2024). In addition to evaluating the stability of dGC capacitance across the light cycle, we address whether voltage-clamp methodology is sufficient to detect relative changes in whole-cell capacitance associated with alterations in the dGC size resulting from acute axotomy or chronic genetically induced overgrowth.

## Materials and Methods

### Animals

**Figure 1.**
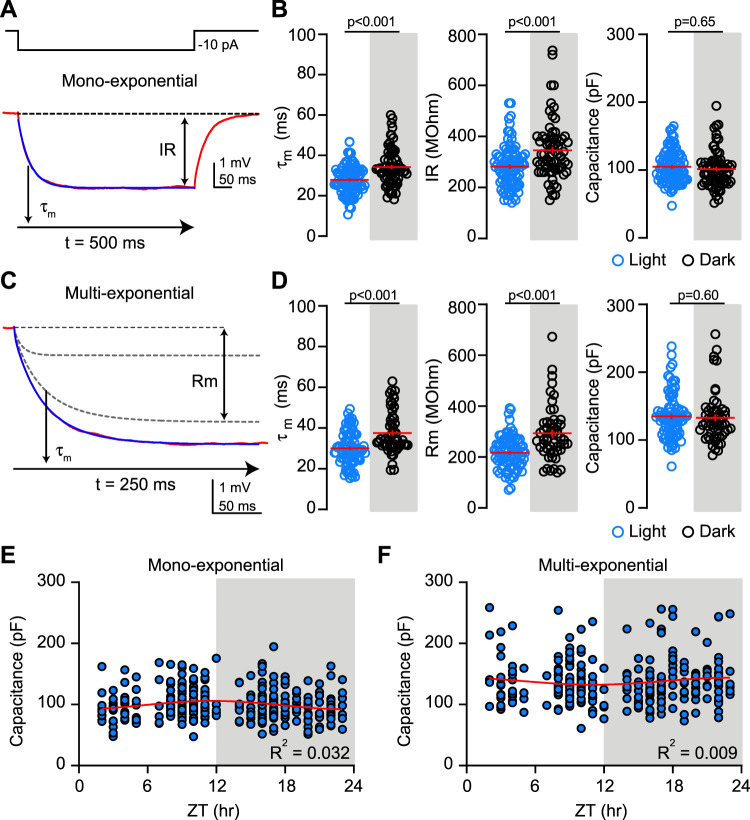
Membrane capacitance remains stable across the light cycle. ***A***, Top, Current-clamp injection (500 ms, −10 pA) used to generate voltage responses. Bottom, Average voltage response (red) analyzed with a monoexponential fit (blue) to measure IR and time constant (*τ*_m_). ***B***, Scatterplots show time constant (left), IR (middle), and capacitance (right) during the light (blue) and dark (black) phases. Mann–Whitney *U* test, *U* = 1,845 (*τ*_m_), *U* = 1,997 (IR), and unpaired *t* test, *t* = 0.45 (capacitance). *n* = 90 (light); 69 (dark). Lines indicate mean ± SEM. ***C***, Same average voltage response as in ***A*** (red) analyzed with a multiexponential fit (blue). The slowest component was used to measure membrane resistance (Rm) and time constant (*τ*_m_). ***D***, Scatterplots show time constant (left), membrane resistance (middle), and capacitance (right) during the light (blue) and dark (black) phases. Mann–Whitney *U* test, *U* = 1,234 (*τ_m_*), *U* = 1,155 (Rm), and unpaired *t* test, *t* = 0.51 (capacitance). *n* = 84 (light); 53 (dark). Lines indicate mean ± SEM. ***E***, ***F***, Dot plots show capacitance measured with mono- (***E***) or multiexponential fit (***F***) averaged in 1 h bins fit with Cosinor function across 24 h. Lights on at ZT0 and off at ZT12. *n* = 2–38 cells per bin.

**Figure 2.**
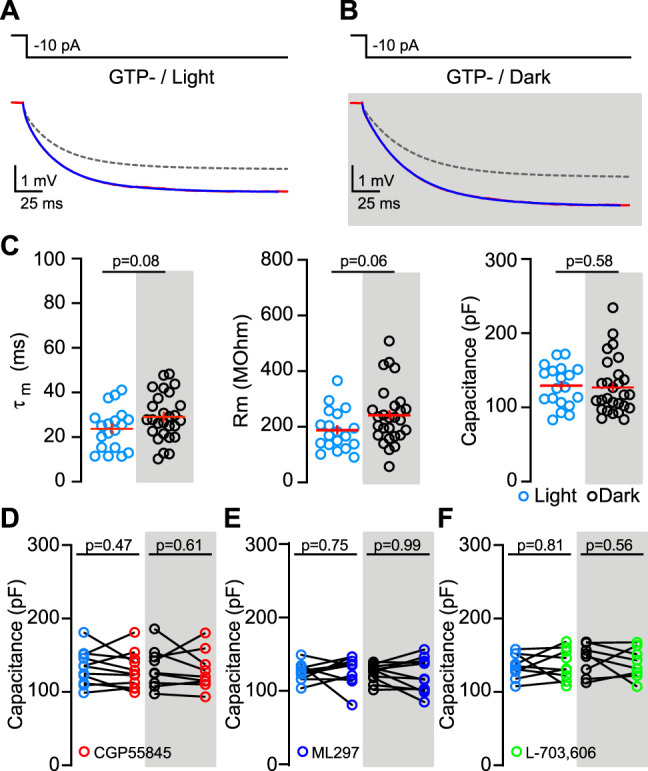
Manipulating circadian-regulated membrane conductances confirms stability of membrane capacitance. ***A***, ***B***, Current injections (500 ms, −10 pA) during light (***A***) and dark (***B***) used to generate voltage responses using an internal pipette solution without GTP (GTP^−^). Multiexponential slowest components are shown with dash lines. ***C***, Scatterplots show time constant (left), membrane resistance (middle), and capacitance (right) during the light (blue) and dark (black) phases in GTP^−^ conditions. Unpaired *t* test, *t* = 1.77 (*τ*_m_); Mann–Whitney *U* test, *U* = 172 (Rm); and Mann–Whitney *U* test, *U* = 231 (capacitance). *n* = 19 (light); 27 (dark). Lines indicate mean ± SEM. ***D***, The scatterplot shows capacitance before and after GABA_B_ inverse agonist CGP55845 (CGP 10 μm) during light and dark. Paired *t* test *t* = 0.75 (light); *t* = 0.52 (dark). *n* = 11 (light); 9 (dark). Analysis of two-way ANOVA shows no differences in dGCs capacitance (*F*_(1,36)_ = 0.59, 0.001, and 0.0001; *p* = 0.59, 0.97, and 0.99 for CGP, recording window and interaction, respectively). ***E***, The scatterplot shows capacitance before and after GIRK1 activator ML297 (10 μm) during light and dark. Paired *t* test *t* = 0.31 (light); *t* = 0.004 (dark). *n* = 12 (light); 10 (dark). Analysis of two-way ANOVA shows no differences in dGCs capacitance (*F*_(1,40)_ = 0.04, 0.22, and 0.03; *p* = 0.84, 0.63, and 0.84 for ML297, recording window and interaction, respectively). ***F***, The scatterplot shows capacitance before and after the NaLCN selective blocker L-703,606 (10 μm) during light and dark. Paired *t* test *t* = 0.24 (light), *t* = 0.60 (dark). *n* = 9 (light); 8 (dark). Analysis of two-way ANOVA shows no differences in dGCs capacitance (*F*_(1,30)_ = 0.05, 0.55, and 0.62; *p* = 0.82, 0.46, and 0.62 for L-703,606, recording window and interaction, respectively).

Mice reported in Gonzalez et al. (2023) included 2- to 5-month-old males and females from colonies of WT C57BL/6J (Jackson Laboratory #000664), PV-Cre (Jackson Laboratory #017320); nNOS-CreERt2 (Jackson Laboratory #014541); Ai32 (Jackson Laboratory #024109), and Ai14 (Jackson Laboratory #007914) mice, all maintained on a C57BL/6J background (Figs. 1, 2). In new experiments we used 6- to 12-week-old mice of both sexes from colonies of WT C57BL/6J (Jackson Laboratory #000664) and Pten^flx/flx^ mice (Jackson Laboratory #006440; B6.129S4-Pten^tm1Hwu^/J), housed in standard cages with *ad libitum* access to food and water and maintained in a 12:12 light/dark (LD) cycle. All procedures were approved by the University of Alabama at Birmingham Institutional Animal Care and use Committee (IACUC) in accordance with the US National Institute of Health Guide for the Care and Use of Laboratory Animals.

### Slice preparation

**Figure 3.**
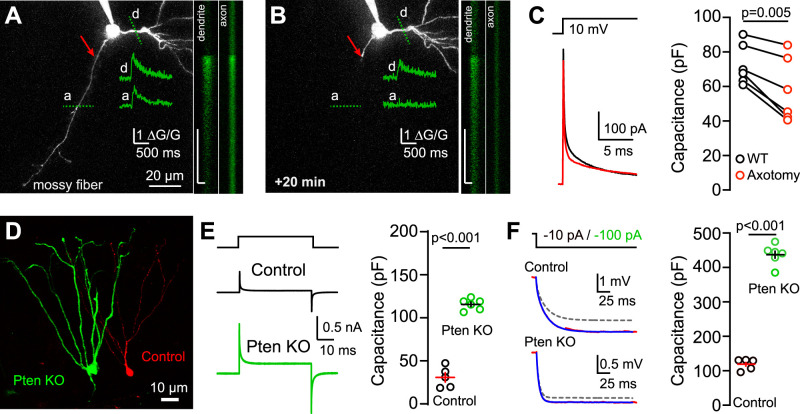
Using capacitance to detect changes in the cell size. ***A–C***, Example of dGC filled with Alexa Fluor 594 before (***A***) and after (***B***) axotomy of the mossy fiber axon (red arrow). Calcium transients elicited by APs were measured by line scans (dotted green lines) across the axon (a) and dendrite (d). Insets on the right of each image show example line scans at each region, with scale bars representing 500 ms (*y* axis) and 1 μm (*x* axis). Axotomy abolishes the AP-induced CaT in the axon and reduces basal axonal fluorescence. ***C***, Left, Current transients evoked by voltage steps (−10 mV) before (black) and after mossy fiber axotomy. Right, scatterplot showing capacitance values. Paired *t* test, *t* = 4.74. *n* = 6. The IR from dGCs held at −70 mV was 403 ± 39 MΩ versus 291 ± 34 MΩ; *p* = 0.008; before and after axotomy, respectively. ***D***, Confocal image of Pten KO (green) and control dGCs (red). ***E***, Left, Average current transients generated by the test pulse (10 mV). Right, Capacitance values obtained by measuring the charge accumulation. Unpaired *t* test, *t* = 14.8. *n* = 5 (control); 6 (Pten). The IR from dGCs held at −90 mV was 114 ± 10 MΩ versus 20 ± 1.3 MΩ; *p* = 0.004; for WT and Pten KOs, respectively. ***F***, Left, Average voltage responses generated by current injection in control (−10 pA) and Pten (−100 pA) dGCs. Multiexponential slowest component shown with dash lines. Right, scatterplot showing capacitance values. Unpaired *t* test, *t* = 21.3. *n* = 5 (control); 6 (Pten). Lines indicate mean ± SEM.

Mice were lightly anesthetized at zeitgeber time (ZT) 5.5 or 11.5 with isoflurane (4%; Fluriso, USP; VetOne) and deeply anesthetized with an intraperitoneal injection of 2,2,2-tribromoethanol (Avertin; Sigma-Aldrich) followed by transcardial perfusion with ice-cold carbogenated cutting solution (5% CO_2_/95% O_2_) containing the following (in mM): 110 choline chloride, 7 MgCl_2_, 3 Na-pyruvate, 2.5 KCl, 1.3 Na-ascorbate, 1.25 Na_2_PO_4_, 0.5 CaCl_2_, 25 d-glucose, and 25 NaHCO_3_. The brain was removed, and 300-μm-thick horizontal slices at an angle that preserves dendritic integrity were prepared using a vibratome (VT1200S, Leica Instruments; Gonzalez et al., 2024). Slices were incubated at 37°C for 30 min in recording solution containing the following (in mM): 125 NaCl, 2.5 KCl, 2 CaCl_2_, 1.25 Na_2_PO_4_, 1 MgCl_2_, 25 d-glucose, and 25 NaHCO_3_ bubbled with 5% CO_2_/95% O_2_. Recordings were started 2 h after slice preparation during a 3 h window (ZT 8–11, light; ZT 14–17, dark) to account for the evolution of intrinsic properties over the circadian cycle, even in vitro (Gonzalez et al., 2023). For Figure 1, *E* and *F*, slices were also prepared at ZT 0 and 18 to cover the entire 24 h cycle, and recordings were pooled in 1 h intervals. Note that Gonzalez et al. (2024) provide a detailed step-by-step protocol for electrophysiological analysis of circadian changes in excitability. For Figure 3, slices were prepared between ZT 4 and 6.

### Electrophysiology

Visually identified mature dGCs in the middle of the granule cell layer were recorded in the whole-cell configuration, avoiding young adult-born and semilunar granule cells. Fire-polished borosilicate glass electrodes (BF150-86-10, Sutter Instrument) with resistance of 3–5 MΩ when filled with intracellular solution were mounted on the headstage of a Multiclamp 700A amplifier (CV-7B, Molecular Devices). Pipettes for current-clamp recordings were filled with the following (in mM): 135 K-gluconate, 10 HEPES, 10 phosphocreatine, 3 KCl, 2 MgCl_2_, 2 Mg-ATP, 0.5 Na-GTP (excluded in GTP^−^ experiments), and 0.1 EGTA, pH 7.3 (310 mOsm). We maintained the intracellular solution on ice to minimize GTP hydrolysis, and we ensured that seal resistance was at least 10 times greater than expected input resistance (IR). In whole-cell configuration, we adjusted bridge balance to compensate for the series resistance (typically 10–15 MΩ), and experiments were discarded if substantial changes in the bridge balance were detected. To measure passive membrane properties including membrane resistance and time constant, we made small hyperpolarizing current injections (10 pA, 500 ms) from resting membrane potential (*I* = 0) and averaged the voltage response of at least 30 sweeps after excluding those with spontaneous events that could compromise the exponential fitting. All recordings were made at 30°C. Currents were sampled at 10 kHz and filtered at 2 kHz (Digidata 1440A; Molecular Devices) using the PClamp 10 software (Molecular Devices).

Axotomy was performed in dGCs using an internal recording solution containing the red-emitting dye Alexa Fluor 594 (25 µM) and green-emitting Ca^2+^ indicator Fluo-5F (100 µM) to allow simultaneous imaging of cell morphology and Ca^2+^ influx before and after axotomy. The 2P excitation was achieved using a Chameleon Vision pulse Ti:Sapphire laser (Coherent) tuned to 810 nm, which is near the peak 2P excitation wavelength for both Alexa Fluor 594 and Fluo-5F. Axotomy was performed ∼25–50 µm from the soma using 5–10 line scans across the axon at 200 mW of power while recording in the current-clamp configuration (Pennock et al., 2023). Action potentials (APs) were induced before and after axotomy using a 200 pA current injection (50 ms) while simultaneously performing line scans (1 kHz) across the axon distal to the cutting location or across the primary dendrite. The loss of Alexa Fluor 594 fluorescence and the absence of AP-induced increase in Fluo-5F fluorescence in the axon was used to confirm successful axotomy, while the continued presence of AP-induced Fluo-5F fluorescence after axotomy in the dendrites was used as an indicator of general cell health. Changes in Fluo-5F fluorescence were analyzed using custom MATLAB scripts as described in Pennock et al. (2023). Changes in capacitance were measured in voltage-clamp configuration by applying voltage steps (+10 mV, 100 ms) before and after axotomy.

### Stereotaxic injections to label newly generated dGCs

Pten^flx/flx^ mice at postnatal day (P)7 were anesthetized with isofluorane and placed into a stereotaxic frame. Bilateral craniotomies were performed at ±1.3 mm lateral and 1.55 mm from lambda. Replication-defective retroviruses based on pRubi (Luikart et al., 2011) were injected 2/2.2/2.3/2.4 mm deep from pia using a 10 μl Hamilton. Retroviral vectors expressing Cre recombinase with a GFP reporter and a Cre recombinase void vector with mCherry reporter were coinjected, resulting in Pten KO dGCs expressing GFP and control dGCs of the same age expressing mCherry (Williams et al., 2015). Final volume of 2 μl of virus was injected at each hemisphere at a rate of 0.3 μl/min (titer, pRubi-GFP-T2A-Cre 9.28 × 10^8^ viral genomes/ml and Red-Rubi 4.6 × 10^8^ viral genomes/ml). Slices containing viral-labeled control and Pten KO dGCs were obtained as described above and recorded in voltage- and current-clamp mode. Pipettes were filled with the following internal solution (in mM): 115 K-gluconate, 20 KCl, 10 HEPES, 10 Na-phosphocreatine, 2 Mg-ATP, 2 EGTA, and 0.3 Na-GTP. Capacitance was measured in voltage clamp by applying voltage steps (+10 mV, 40 ms) and in current clamp by applying hyperpolarizing current injections (10 pA, 500 ms). All recordings were made at 32°C. Currents were sampled at 40 kHz and filtered at 2 kHz (Multiclamp 700B; Molecular Devices) using Axograph X software (Axograph.com).

### Immunohistochemistry

At 60 d postinjection, brains were sliced with Leica VT1000S after perfusing with PBS-Sucrose (4%) followed by 4% paraformaldehyde. The 50 µm slices were prepared and stained with chicken anti-GFP (1:3,000 dilution, Abcam #13970) and mouse anti-mCherry (1:3,000 dilution, Takara Bio #632543). Sections were treated with secondary antibodies of Alexa Fluor 488 goat anti-chicken (1:200 dilution, Jackson ImmunoResearch Laboratories #103-545-155) and Cy3 donkey anti-mouse (1:200 dilution, Jackson ImmunoResearch Laboratories #715-165-150). Sections were imaged with Zeiss LSM510 laser-scanning confocal microscope using a 40× oil objective lens.

### Quantification and statistical analysis

We used multiple approaches to assess the capacitance of GCs. For current-clamp recordings, the membrane time constant was measured by fitting the average voltage response to a small hyperpolarizing current (10 pA, 500 ms) using monoexponential or multiexponential equations. We originally used a monoexponential fit over the entire 500 ms current step to measure the time constant (*τ*_m_) and the amplitude at the end of step to calculate the IR (Gonzalez et al., 2023). This generated good fits with a correlation coefficient of >0.95 in most GCs (0.995 ± 0.003). We used the time constant to calculate membrane capacitance *C*_m_ following the relationship *C*_m_ = *τ*_m_ / IR. We reanalyzed most of the original dataset using multiexponential fitting following the approach of Severin and collaborators using the Levenberg–Marquardt algorithm of the Clampfit analysis software in the pClamp package (Molecular Devices), with a precision of 10^−6^ (Golowasch et al., 2009; Severin et al., 2024). Voltages were fit over a 250 ms window using a two- or three-term exponential function, and values of the slowest component were used to calculate the capacitance. With this method, membrane resistance is derived from the amplitude of the slowest component and thus is less than the IR of the cell (Golowasch et al., 2009; Severin et al., 2024).

To calculate the capacitance in voltage-clamp recordings, we integrated the area under the transient currents generated by small voltage steps after subtracting the steady-state current. The charge (*Q*) was then divided by the size of the voltage step (*V*) to calculate capacitance following the relationship *C*_m_ = *Q* / *V* (Golowasch et al., 2009; Taylor, 2012). To also calculate the fast and the slow components of the capacitance in the axotomy experiments, we cropped the decay phase of the current transients evoked by the test pulse (100 ms, 10 mV) and imported them into Prism where they were fit with a two-phase decaying exponential function. The amplitude and time constants (*τ*) of the fast and slow component of the exponential fit were used to calculate *C*_m_ fast and slow according to as follows:

$$C_{m}=\frac{A\;*\;\tau }{V_{\mathrm{step}}},$$

where *A* is the amplitude of the fast or slow component, *τ* is the time constant of the fast or slow component, and *V*_step_ is the voltage step (+10 mV in this case).

Data are expressed as mean ± SEM. Outcome parameters were tested for normality. Group comparisons used two-way ANOVA, Mann–Whitney *U* test, two-tailed unpaired or two-sample paired *t* test with statistical significance at *p* < 0.05. Rhythmic comparison of capacitance was calculated using Cosinor analysis with the following equation:

f(t)=Mesor+A*Cos[(2πt/T)+Acrophase],

where Mesor (acronym for middle estimating statistic of rhythm) is the mean of the oscillation; *A* is the amplitude (peak-to-trough difference); *T* is the period (24 h); Acrophase is the timing of the cosine maximum; *t* is a time point; and *R*^2^ is the resulting statistic that measures the percentage variance accounted for by the 24 h approximating waveform. Statistical analyses were performed using Prism 10.4 (GraphPad Software).

## Results

### Membrane capacitance remains stable across the light cycle

To address whether neuronal capacitance changes across the light cycle, we reanalyzed data from Gonzalez et al. (2023) where we measured the IR and membrane time constant (*τ*_m_) of dGCs during the light (ZT 8–11) and dark phase (ZT 14–17) using voltage changes in response to small hyperpolarizing current injections (10 pA, 500 ms). We originally used a single term exponential fit to determine the time to reach ∼63% of the final current corresponding to the time constant, and we used the amplitude of the steady-state current to calculate the IR (Fig. 1*A*). As we previously reported, there is a significant difference in both the time constant and IR of dGCs during the light and the dark. During the dark, there is a slower time constant (34.2 ± 1.1 ms vs 27.8 ± 0.7 ms; *p* < 0.001) and higher IR (345 ± 13 MΩ vs 281 ± 8 MΩ; *p* < 0.001; Fig. 1*B*). Using these values to calculate the capacitance (*C*_m_ = *τ*_m_/IR) suggests there is no difference in dGC capacitance at these time points (103.3 ± 2.6 pF vs 101.1 ± 3.3 pF; *p* = 0.65; Fig. 1*B*, right).

To avoid potential inaccurate quantification of capacitance due to the nonisopotential properties of neurons with complex morphology, we reanalyzed the voltage responses using multiexponential fits. Neurons with larger and/or more complex morphology exhibit membrane potential changes that are best fit by a series of exponential terms that reflect current flow between nonisopotential electrical compartments (Golowasch et al., 2009). We therefore also fit many of the average voltage traces using an exponential function with two or three terms and used the parameters from the slowest component to calculate membrane capacitance (Fig. 1*C*; see Materials and Methods). To replicate the methods of Severin et al. (2024), we fit the voltage response over the first 250 ms rather than 500 ms. This approach also reveals a slower time constant (37.4 ± 1.4 ms vs 30 ± 0.9 ms; *p* < 0.001) and higher membrane resistance during the dark (295.0 ± 14.7 MΩ vs 216.8 ± 7.0 MΩ; *p* < 0.001; Fig. 1*D*). Using these values from the multiexponential analysis resulted in higher values of calculated membrane capacitance compared with the monoexponential analysis (compared with Fig. 1*B*, right; 132.8 ± 5.2 pF in the dark and 136 ± 4.2 pF in the light, two-way ANOVA; *F*_(1,277)_ = 72.40; *p* < 0.0001), supporting the idea that the multiexponential fitting approach provides a more accurate estimation of the total cell capacitance (Golowasch et al., 2009; Taylor, 2012). However, it also showed that the capacitance of dGCs was similar across the light and dark phase (*p* = 0.60; Fig. 1*D*, right). Additionally, two-way ANOVA analysis showed no difference in capacitance between dGCs in male and female mice with average values of 133 ± 3.5 pF versus 132 ± 2.8 pF, respectively.

Nonetheless, comparing dGC membrane properties during only two time windows could miss a daily cycle. We previously made recordings at 1 h intervals across the light cycle to show that dGC resting membrane potential, IR and time constant exhibit a continuous oscillation (Gonzalez et al., 2023). Using this dataset, we used both approaches of calculating capacitance to assess whether it varies across the entire light cycle. When capacitance values are pooled in 1 h bins, the monoexponential approach revealed no significant rhythmicity using Cosinor analysis (*R*^2^ = 0.032; mesor = 98.9 and amplitude = 6.9 pF; *F*_(2,325)_ = 1.37; *p* = 0.12; Fig. 1*E*). Likewise, values of membrane capacitance calculated using the multiexponential fits were stable (*R*^2^ = 0.009; mesor = 137.4 and amplitude = 4.7 pF; *F*_(2,276)_ = 1.28; *p* = 0.27; Fig. 1*F*). Together these results support the notion that dGC capacitance is constant despite rhythmic changes in other membrane properties.

### Manipulating circadian-regulated membrane conductances confirms stability of membrane capacitance

Mature dGCs exhibit constitutive GABA_B_ receptor-coupled G–protein inwardly rectifying potassium (GIRK) channel activity that contributes to low IR and hyperpolarized resting membrane potential (Gonzalez et al., 2018). Constitutive GIRK channel activity is absent during the dark phase, while sodium leak channel (NaLCN) activity is present during the dark phase but not during the light phase. As both GIRK and NaLCN channel activity depend on G-protein signaling, removing GTP from the intracellular solution (GTP^−^) abolishes the differences in passive membrane properties between light and dark phases (Gonzalez et al., 2018, 2023). To address the possibility that GTP-dependent changes in membrane resistance could mask a daily cycle of dGC capacitance, we used the multiexponential fitting approach to reanalyze data during the light and dark phases from recordings without GTP in the pipette (GTP^−^ conditions; Fig. 2*A*,*B*). As expected, under these conditions, there was no significant difference in the time constant (23.7 ± 2.1 ms vs 29.0 ± 2.0 ms; Fig. 2*C*, left) nor the membrane resistance (186.5 ± 16.8 MΩ vs 240.7 ± 20.1 MΩ; Fig. 2*C*, middle). Likewise, calculations of membrane capacitance revealed no significant difference (128.8 ± 6.3 pF vs 128.0 ± 7.3 pF; Fig. 2*C*, right). Furthermore, in the absence of intracellular GTP, the capacitance values remain comparable with those recorded in GTP-containing (GTP^+^) conditions (all LD data pooled together GTP^+^ vs GTP^−^ 134.1 ± 3.1 pF vs 126 ± 4.4 pF; *p* = 0.2). Thus, there were no differences in membrane capacitance across the time of day even when we prevent differences in membrane resistance by excluding GTP from the recording pipette.

As the presence of GTP was required to detect GIRK and NALCN activity that contribute to daily cycles of dGC membrane resistance, we further tested whether individual block of these channels would reveal differences in capacitance. The inverse GABA_B_ receptor agonist CGP55845 (CGP; 10 μm) increases membrane resistance during the light phase but not the dark (Gonzalez et al., 2018, 2023). However, multiexponential analysis revealed that CGP55845 did not alter membrane capacitance either during the light (133.5 ± 7.1 pF vs 129.1 ± 7.4 pF) or the dark (133.1 ± 9.1 pF vs 128.9 ± 8.9 pF; Fig. 2*D*). Direct activation of GIRK channels using the activator ML297 (10 μm) reduces membrane resistance to similar levels during the light and dark phase (Gonzalez et al., 2023). However, multiexponential analysis revealed that ML297 did not alter capacitance during the light (124.8 ± 3.3 pF vs 126.7 ± 5.4 pF) nor the dark (125.3 ± 3.8 pF vs 125.3 ± 6.6 pF; Fig. 2*E*). Finally, blocking NaLCN with L-703,606 (10 μm) had no effect during the light but hyperpolarized dGCs during the dark (Gonzalez et al., 2023). Yet again, membrane capacitance of dGCs remains unaltered either during the light (135.6 ± 5.6 pF vs 137.4 ± 7.1 pF) or the dark (144.1 ± 7.5 pF vs 139.1 ± 7.4 pF; Fig. 2*F*). Together these results support the conclusion that a daily cycle of dGC membrane time constant results from changes in membrane resistance rather than capacitance. These results reinforce the idea that capacitance is a stable biophysical property that does not oscillate over the time of day.

### Using capacitance to detect changes in the cell size

Given that we did not detect significant changes in capacitance across the time of day, we sought to assess the sensitivity of our measures for the membrane area. Despite the caveats in accurately measuring the capacitance of nonisopotential neurons with complex anatomy, transient currents from voltage steps or single exponential fits of voltages are routinely used as relative indicators of the cell size. For example, these measures have been used to track developmental increases in dGC dendritic length and complexity in the developing and adult brain (Ambrogini et al., 2004; Esposito et al., 2005; Laplagne et al., 2007; Mongiat et al., 2009; Williams et al., 2015; Kennedy et al., 2024). Thus, we wondered whether these approaches nonetheless provide relatively precise comparisons of the cell size. First, we tested whether the least sensitive method using voltage clamp is sufficient to detect changes in the cell size resulting from acute axotomy. We made simultaneous electrophysiological recordings and two-photon imaging of dGCs filled with Alexa Fluor 594 and the Ca^2+^ indicator Fluo-5F to image cell morphology and monitor AP-induced Ca^2+^ influx in the axonal and dendritic compartments. Baseline current responses to a 10 mV voltage step, as well AP-induced Ca^2+^ influx, were measured before axotomy (Fig. 3*A*,*C*). Axotomy was then achieved using 5–10 high-powered line scans perpendicular to the axon (Mejia-Gervacio et al., 2007; Pennock et al., 2023). After 20 min, we verified successful axotomy and cell viability by selective loss of AP Ca^2+^ influx in the axon with unchanged Ca^2+^ influx in the dendritic tree (Fig. 3*B*). In small neurons with large axons like cerebellar MLIs (i.e., *C*_m_ ∼30 pF), axotomy reduces a large but slow component of capacitance current (Mejia-Gervacio et al., 2007; Pennock et al., 2023). In dGCs with a larger somatodendritic area, we found the capacitance current also showed two components, with the slow component representing ∼90% of the total capacitance. Axotomy modestly reduced the fast (6.9 ± 1.0 pF vs 6.0 ± 0.7 pF; *p* = 0.02) and markedly reduced the slow component (58.2 ± 3.7 pF vs 46.7 ± 5.5 pF; *p* = 0.007; not shown), resulting in an average reduction in the total capacitance of ∼20% (65.2 ± 4.4 pF vs 52.8 ± 6.2 pF; *p* = 0.004; Fig. 3*C*, left). We also measured the capacitance by integrating the area under the transient current (after subtracting the steady-state current) to obtain the total charge (*Q*) and then used the size of the voltage step (*V*) to calculate capacitance following the relationship *C*_m_ = *Q*/*V* (Golowasch et al., 2009; Taylor, 2012). This common method likewise revealed that axotomy significantly reduces the capacitance by ∼20% (73.0 ± 4.8 pF vs 58.2 ± 7.6 pF; *p* = 0.005; Fig. 3*C*, right). Thus, although voltage-clamp measurements provide smaller capacitance values compared with current-clamp measurements (see above), these results confirm the capacity of the voltage-step method to discriminate acute modifications in the cell size.

Having confirmed that transient currents from voltage steps can detect acute changes in the dGC size, we next asked whether they provide similar relative measures of the cell size as voltage responses using a model of genetic-induced overgrowth. Genetic deletion of phosphatase and tensin homolog on chromosome 10 (Pten) is associated with a marked increase in the dGC size and capacitance resulting from somatodendritic hypertrophy (Luikart et al., 2011; Williams et al., 2015). We compared capacitance measures from voltage-clamp and current-clamp recordings of mCherry (control) and GFP-expressing Cre-dependent Pten KO (Pten KO) dGCs at 50 d after retroviral infection at P7. Comparing the transient currents from 10 mV steps revealed a large increase in the capacitance of Pten KO dGCs (115.6 ± 2.6 pF) compared with control dGCs (30.7 ± 5.4 pF; *p* < 0.001; Fig. 3*E*). In the same cells, we calculated the capacitance using multiexponential fits to small negative current injections. This also revealed a large increase in capacitance of Pten KO dGCs (437.4 ± 12.1 pF) compared with control dGCs (118.8 ± 7.3 pF; *p* < 0.001; Fig. 3*F*). While the absolute values of these measures illustrate that the current measures significantly underestimate capacitance, the relative increase was similar (376 ± 8.5% vs 368 ± 10.2%; *p* = 0.56). The current-clamp recordings also revealed significant reductions in the time constant and membrane resistance in Pten KO dGCs (25.5 ± 1.6 ms vs 9.1 ± 1.3 ms; 218.2 ± 20.1 MΩ vs 20.6 ± 2.6 MΩ; WT vs Pten KO, respectively), as previously reported (Luikart et al., 2011; Williams et al., 2015). Together these results indicate that both voltage-clamp and current-clamp methods can provide comparable relative estimates of changes in dGC size.

## Discussion

Here we show that dGCs capacitance remains stable across the circadian cycle. We use a large pool of current-clamp recordings where we compare capacitance measures obtained using a monoexponential and a multiexponential fitting approach. In both cases, we find that the membrane resistance and time constant change over the light cycle. However, we did not find any rhythmicity of membrane capacitance. Pharmacological manipulations that differentially alter membrane resistance across the light cycle also did not unmask differences. Furthermore, we show that widely used measures of capacitance based on current transients in response to voltage steps are sensitive to changes in the cell size and can provide similar relative measures despite generating smaller absolute values compared with current-clamp recordings. Thus, various methods of measuring capacitance as an estimate of the cell size or membrane area can be useful despite differences in accuracy (Golowasch et al., 2009).

A recent study proposed that fluctuations in cell excitability across the light cycle are due to daily changes in membrane capacitance, challenging the assumption that neuronal membrane capacitance provides a stable measure of the cell size (Severin et al., 2024). The hippocampal cell subtype used in that study is the dGC, which we previously used to report circadian oscillation in passive and active membrane properties (Gonzalez et al., 2023). The relative change in membrane capacitance reported across the light cycle is on the order of the change that occurs during dGC morphological maturation over the course of several weeks of development (Tyzio et al., 2003; Kennedy et al., 2024). We aimed to elucidate whether our dataset of dGC recordings likewise revealed a change in capacitance across the light cycle that could contribute to excitability, but we did not find changes regardless of different analysis methods (Golowasch et al., 2009). Our values of time constant and membrane resistance are consistent with those reported previously for mature dGCs by ourselves and others (Spruston and Johnston, 1992; Schmidt-Hieber et al., 2007; Kennedy et al., 2024), with a constant value of membrane capacitance in the range of ∼130 pF. It is possible that various technical differences such as seal tightness, cell age, temperature, and membrane potential could contribute to different outcomes. During our experiments, we noted that voltage-clamp measures were sensitive to the seal resistance and the command potential, making these variables important to control when comparing cell populations or conditions.

Membrane conductivity and capacitance govern the intrinsic excitability of neurons (Hodgkin and Huxley, 1952). Membrane conductivity can be transiently switched by electrical, optogenetic, and pharmacological manipulations. We use pharmacology to remove circadian regulation of membrane resistance to test whether changes in dGCs capacitance can be unmasked. Previously, we linked constitutive activation of GIRK channels to developmental stage (Gonzalez et al., 2018) and circadian regulation of passive properties together with NaLCN (Gonzalez et al., 2023). However, manipulating these conductances to modulate dGC excitability also did not change our estimates of membrane capacitance across the light cycle. While physiological processes such as morphological development or myelinization modulate capacitance, changes beyond these contexts are typically associated with pathological conditions, such as overgrowth in autism (Luikart et al., 2011), degeneration in epilepsy (Isokawa, 1996), activation of astrocytes in inflammation (Karpuk et al., 2012), and degradation in perineuronal nets near brain tumors (Tewari et al., 2018). While various methods of measuring capacitance are more or less accurate in nonisopotential cells with complex architecture (Spruston, 2008; Golowasch et al., 2009; Norenberg et al., 2010; Taylor, 2012), our results suggest that multiple approaches can discern relative changes of dGC capacitance associated with large changes in the cell size such as hypertrophy in the Pten autism model (Luikart et al., 2011) or more subtle variations after acute axotomy (Mejia-Gervacio et al., 2007; Pennock et al., 2023). As predicted, voltage-clamp measurement provided smaller estimates of capacitance compared with current-clamp measurements, supporting that the latter provide more accurate total membrane capacitance values. This idea is also supported by the necessity of adding dendritic spines in simulations in order to reproduce experimental measurements (Schmidt-Hieber et al., 2007). Our finding that voltage responses are relatively well-fit with monoexponential functions is consistent with the idea that dGCs are electrotonically compact (Spruston and Johnston, 1992; Schmidt-Hieber et al., 2007).

There is a long-standing literature showing that neurons throughout the hippocampus exhibit daily oscillations in cellular properties including intrinsic excitability and synaptic plasticity (Harris and Teyler, 1983; Liu et al., 2000; Chaudhury et al., 2005; McCauley et al., 2020; Goode et al., 2022). We found that daily changes in intrinsic excitability contribute to a lower probability of cortical-evoked dGC spiking in slices during the light phase (Gonzalez et al., 2023), in line with the daily cycle of cortical-evoked population spikes reported in vivo (Barnes et al., 1977). This oscillation is dependent on the dGC-intrinsic molecular clock, as conditional deletion of *Bmal1* in dGCs led to persistent high excitability that phenocopied the dark phase despite no change in systemic circadian rhythmicity (Gonzalez et al., 2023). Such local clock control of neuronal physiology provides a direct link between molecular machinery and membrane excitability, extending circadian regulation beyond the suprachiasmatic nucleus. The alignment of cell-intrinsic excitability states with an organism's systemic circadian cycles may optimize hippocampal function by enhancing memory encoding during the active phase while promoting signal filtering and consolidation during rest. Circadian regulation of performance on hippocampal-dependent memory tasks is documented across many species, and genetic misalignment of local and systemic oscillations by cell-type–specific deletion of molecular clock components impairs hippocampal functions (Snider et al., 2018; Hasegawa et al., 2019). Identifying links between intrinsic and systemic oscillators and their respective roles in controlling neuronal properties is an important future goal for understanding how static brain circuits flexibly give rise to diverse patterns of activity across the circadian cycle.

Disruption of appropriate circadian regulation is increasingly implicated in impaired cognitive performance and susceptibility to network instability in pathological conditions including epilepsy and neurodegenerative disorders (Huang et al., 2024). The dentate gyrus is particularly important in the pathophysiology of temporal lobe epilepsy, as it serves as a key hub for suppressing propagation of epileptiform activity from the cortex (i.e., the “dentate gate”; Krook-Magnuson et al., 2015). For example, the frequency of seizure activity in rodent models of temporal lobe epilepsy varies across the time of day and correlates with dentate gyrus excitability (Matzen et al., 2012). Furthermore, *Bmal1* expression in the dentate gyrus is reduced in the chronic phase of experimentally induced epilepsy, and conditional deletion of *Bmal1* in naive mice reduces the threshold for seizure induction (Wu et al., 2021). Together these results suggest that *Bmal1*-dependent suppression of dGC excitability plays a role in maintaining the dentate gate in both pathological and physiological conditions. Our analysis suggests that the daily cycle of dGC excitability results from changes in membrane resistance rather than capacitance, providing insight into the biophysical mechanisms underlying circadian modulation of neuronal excitability.

## Synthesis

Reviewing Editor: Harald Sontheimer, University of Virginia Decisions are customarily a result of the Reviewing Editor and the peer reviewers coming together and discussing their recommendations until a consensus is reached. When revisions are invited, a fact-based synthesis statement explaining their decision and outlining what is needed to prepare a revision will be listed below. The following reviewer(s) agreed to reveal their identity: Bhanu Tewari.

This paper adds some interesting observations to the recently sparked debate on membrane capacitance, which is generally considered a stable property which is mainly governed by the size of the cell. However, several recent studies have reported changes in capacitance in physiology and pathology, particularly in dentate granule cells (dGCs). According to them, the membrane capacitance oscillations are due to changes in the membrane time constant at the light and dark phases of the light-dark cycle while membrane resistance remains largely stable. In the current manuscript, Gonzalez et al assessed their data from previous studies and compared it with the findings of Severin et al 2024. The authors computed the input resistance, time constant, and capacitance of dGCs in the dark and light phases similar to Severin et al 2024 and confirmed the daily oscillations in the time constant and membrane resistance but did not see a consequent change in membrane capacitance. These data partly recapitulate the findings of Severin et al 2024 however attribute the changes in time constant to the membrane resistance rather than the membrane capacitance as reported by Severin et al 2024. The bottom line of the paper is that the capacitance remains stable but the membrane resistance and time constant changes with the light-dark cycle. Overall, the study is focused, methodically sound, and adds one more set of data to enrich the debate on membrane capacitance.

However the following concerns should be addressed to make this highly technical paper beneficial for a broad readership.

1. From a biophysical perspective, these are exciting observations, however authors must add some insight on how these changes can govern the synaptic physiology and overall excitability of the hippocampal/brain circuits.

2. Are there any pathologies/or any hippocampal/brain circuits whose homeostasis is maintained by these oscillations? Please discuss.

3. Since capacitance is generally derived from the membrane resistance and time constant it's a bit puzzling that authors don't see a change in capacitance despite seeing changes in both membrane resistance and time constant. A brief clarification would help.

4. The methodical details are well written, however considering the importance of the voltage or current clamp method/s used to compute capacitance, it would be useful to provide a step-by-step analysis protocol as a supplement.

5. The mono-exponential fit in Fig. 1A looks perfect. I find it hard to achieve such a perfect curve fit with monoexponential decay when recording from a neuron in brain slices. How good was the fitting (correlation coefficient)?

6. Fig. 3 B: The confirmation of axotomy by the loss of AP-induced fluorescence in the axon is not clear to me. The green traces seem to be from the dendritic domain. Can authors show the before and after axotomy traces from the axon? Also, the authors need to present the corresponding Rm and tau values of Fig. 3C, E, and F.

## Author Response

We thank the reviewers for constructive comments that helped to enhance the clarity and significance of our findings. We made edits to the text and figures to address all their concerns. We also made edits in the statistical reporting to make sure that we consistently reported stats for T-tests or Mann Whitney U tests for parametric or non-parametric data sets, respectively, and we updated all figures to include the exact p values. All changes are highlighted in the marked-up version, and they did not alter any of the conclusions.

1. From a biophysical perspective, these are exciting observations, however authors must add some insight on how these changes can govern the synaptic physiology and overall excitability of the hippocampal/brain circuits.

To provide more insight into how these changes potentially govern excitability and function in the normal brain, we included an additional paragraph to the discussion, beginning on page 17.

2. Are there any pathologies/or any hippocampal/brain circuits whose homeostasis is maintained by these oscillations? Please discuss.

To provide more insight into how dysregulation of circadian excitability potentially contributes to pathology, we included an additional paragraph discussing the potential role of dentate excitability and circadian regulation in temporal lobe epilepsy, beginning on page 18.

3. Since capacitance is generally derived from the membrane resistance and time constant it's a bit puzzling that authors don't see a change in capacitance despite seeing changes in both membrane resistance and time constant. A brief clarification would help.

It is correct that the membrane capacitance is related to resistance and time constant by : tm = CmRm. Our results show that the daily change in experimentally measured tm results from a change in Rm (experimentally measured) whereas Cm (calculated) does not change.

In the Methods we have further clarified potential misunderstandings by better explaining the different approaches to measure Cm. In current-clamp recordings we measure the membrane time constant and membrane resistance and then calculate the membrane capacitance. In voltage clamp, membrane capacitance is estimated by the current transients generated by the voltage step (i.e. the capacitance transients). As detailed extensively in past literature, the voltage clamp approach has limitations as it only assays capacitance from the "well-clamped" portion of the cell (i.e. Golowasch et al., 2009). There are also multiple ways of analyzing the current transients obtained by voltage steps. A common approach is to integrate the area under the transient to obtain the charge (Q). Then the charge (Q) is used to estimate capacitance using the relationship Cm = Q / V. Another approach is to fit the decay phase of the transients with two- (or more) exponential functions to estimate the Cm of each component according to C_m=(A*)/V_step . Our results confirm that 1) current clamp measures provide larger estimates of Cm (Golowasch et al., 2009); 2) even though different analysis approaches generate different Cm values, all were able to detect relative changes in dGC size; and 3) the change in dGC time constant across the light cycle results from changes in Rm rather than Cm.

4. The methodical details are well written, however considering the importance of the voltage or current clamp method/s used to compute capacitance, it would be useful to provide a step-by-step analysis protocol as a supplement.

We have enhanced the clarity and included more step-by-step details in the methods, but we don't think a supplement is needed because we followed the protocols described by Golowasch et al., J Phys (2009). This prior work provides an in-depth step-by-step protocol and technical explanation along with modeling to explain the differences between each method, in a manner that is beyond the scope of our work. We now also highlight our published STARS protocol that provides an exhaustive step-by-step protocol for electrophysiological measurements of circadian regulation of excitability (Gonzalez et al., STAR Protocols, 2024).

5. The mono-exponential fit in Fig. 1A looks perfect. I find it hard to achieve such a perfect curve fit with monoexponential decay when recording from a neuron in brain slices. How good was the fitting (correlation coefficient)? The correlation coefficient of the mono-exponential fits shown in Figure 1 had an average value of 0.995 with a standard deviation of 0.003 (now included in the Methods). We optimize the fitting in several ways. We only proceed with recordings when the initial seal resistance is at least ten times greater than the expected input resistance (> 3GOhm). Once in whole-cell configuration, we acquire 50 sweeps per cell and we excluded individual traces with large spontaneous events that could interfere with the fitting (averaging at least 30 sweeps). We have now clarified these points in the Materials and Methods section.

More importantly, whether the voltage response fits well with a mono-exponential component depends on the electro-morphological characteristics of the particular cell. The high quality of mono-exponential fits in dentate granule cells is consistent with past work showing they are electrotonically compact (i.e. Schmidt-Hieber, 2007). Neurons with larger and/or more complex morphology or other properties that make them less electrotonically compact are better fit by a series of exponential terms that reflect current flow between non-isopotential compartments (Golowasch et al., 2009). We have clarified this point.

6. Fig. 3 B: The confirmation of axotomy by the loss of AP-induced fluorescence in the axon is not clear to me. The green traces seem to be from the dendritic domain. Can authors show the before and after axotomy traces from the axon? Also, the authors need to present the corresponding Rm and tau values of Fig. 3C, E, and F.

Thank you for requesting clarification in this figure. It is correct that the green traces are from the dendritic domain. As requested, we added before and after traces from axonal line scans to more clearly illustrate the absence of AP-induced Ca2+ transient in the axon after axotomy, while dendritic Ca2+ transients remain intact. We now present the Ca2+ transients as ΔG/G rather than ΔG/R. This is because both indicators dissipate in the cut axon (as shown in the images and line scans), so the ratio ΔG/R is no longer meaningful in the cut axon.

As requested, we have added the Rm and tau values in the result section for Figure 3F (page 14). For Figures 3C and 3E, capacitance was measured from the charge of the transient current generated by voltage steps, as we cannot measure the membrane time constant (i.e. the voltage is "clamped"). As the Cm value calculated in voltage clamp is an underestimate, we don't think it makes sense to calculate tau from the equation tm = CmRm. Nevertheless, following the suggestion, we have included the corresponding input resistance values in the legend of Figure 3C and 3E (page 21).
